# CspC regulates the expression of the glyoxylate cycle genes at stationary phase in *Caulobacter*

**DOI:** 10.1186/s12864-015-1845-1

**Published:** 2015-08-27

**Authors:** Juliana S. Santos, Carolina A. P. T. da Silva, Heloise Balhesteros, Rogério F. Lourenço, Marilis V. Marques

**Affiliations:** Departamento de Microbiologia, Instituto de Ciências Biomédicas, Universidade de São Paulo, Av. Prof. Lineu Prestes 1374, 05508-000 São Paulo, SP Brazil; Departamento de Bioquímica, Instituto de Química, Universidade de São Paulo, São Paulo, SP Brazil

**Keywords:** *Caulobacter crescentus*, Stationary phase, CspC, Glyoxylate cycle

## Abstract

**Background:**

The Cold Shock proteins are RNA binding proteins involved in various cellular processes, including adaptation to low temperature, nutritional stress, cell growth and stationary phase. They may have an impact on gene expression by interfering with RNA stability and acting as transcription antiterminators. *Caulobacter crescentus cspC* is an essential gene encoding a stationary phase-induced protein of the Cold Shock Protein family and this work had as goal investigating the basis for the requirement of this gene for survival at this phase. In this work we investigate the role of CspC in *C. crescentus* stationary phase and discuss the molecular mechanisms that could be involved.

**Results:**

The expression of *cspC* increased significantly at stationary phase in complex media and in glucose depletion, indicating a putative role in responding to carbon starvation. Global transcriptional profiling experiments comparing *cspC* and the wild type strain both at exponential and stationary phases as well as comparing exponential and stationary phase in wild type strain were carried out by DNA microarray analysis. The results showed that the absence of *cspC* affected the transcription of 11 genes at exponential phase and 60 genes at stationary phase. Among the differentially expressed genes it is worth noting those encoding respiratory enzymes and genes for sulfur metabolism, which were upregulated, and those encoding enzymes of the glyoxylate cycle, which were severely downregulated in the mutant at stationary phase. mRNA decay experiments showed that the *aceA* mRNA, encoding isocitrate lyase, was less stable in the *cspC* mutant, indicating that this effect was at least partially due to posttranscriptional regulation. These observations were supported by the observed arrested growth phenotype of the *cspC* strain when grown in acetate as the sole carbon source, and by the upregulation of genes for assimilatory sulfate reduction and methionine biosynthesis.

**Conclusions:**

The stationary phase-induced RNA binding protein CspC has an important role in gene expression at this phase, and is necessary for maximal expression of the glyoxylate cycle genes. In the case of *aceA*, its downregulation may be attributed to the shorter half-life of the mRNA in the *cspC* mutant, indicating that one of the possible regulatory mechanisms is via altering RNA stabilization.

**Electronic supplementary material:**

The online version of this article (doi:10.1186/s12864-015-1845-1) contains supplementary material, which is available to authorized users.

## Background

Cold shock proteins (CSPs) have been studied in several bacteria, and were proposed to have a role as RNA chaperones that prevent the formation of secondary structures on RNAs, favoring translation of mRNAs and also changing their turnover rates [1]. These small proteins have an RNA binding domain that is called the Cold Shock Domain (CSD), but since then it was realized that not all the *csp* genes are induced by cold shock [2]. Bacterial strains usually have many *csp* paralogs, and as common pattern some of them are induced by cold shock and some are induced at stationary phase [3]. In *E. coli*, the CspA protein was shown to promote transcriptional activation, while CspC and CspE are involved in cell division and possibly in chromosome condensation [4, 5]. *E. coli* CspE displays a transcription antitermination activity that depends on its ability to melt the nucleic acid double strand, and is essential for adaptation to low temperature [6, 7]. CspD is induced upon entry into stationary phase and acts as an inhibitor of DNA replication [8]. The effect of mutations in *csp* genes in global gene expression was evaluated in response to cold shock [9], and at normal temperature [7]. However, the extent of CSP action in gene expression during stationary phase remains mostly unknown.

Little is known about these proteins in the alpha subclass of Proteobacteria, but amino acid sequence alignments showed that in some CSPs occurs a duplication of the CSD, not found so far in other groups [10]. The alpha-proteobacterium *Caulobacter crescentus* has in its genome four genes encoding proteins containing CSDs [10]. The expression of *cspA* and *cspB* is induced upon cold shock, while *cspB*, *cspC* and *cspD* are induced during stationary phase, and CspC and CspD proteins contain two CSDs [10, 11]. The cold induction of *C. crescentus cspA* and *cspB* is mainly a result of post-transcriptional regulation, while *cspD* induction at stationary phase is regulated by the two-component system SpdR/SpdS and ppGpp [12, 13].

*C. crescentus* undergoes drastic changes during stationary phase to long periods, assuming an elongated helical morphology and showing increased resistance to several stress conditions [14]. In order to establish the roles of CspC and CspD in *C. crescentus* adaptation to stationary phase, null mutant strains were obtained [11]. Although saturating transposon mutagenesis experiments predicted the *cspC* gene to be essential in *C. crescentus* [15], we were able to obtain a Δ*cspC::*Ω*Spec*^*R*^ mutant strain after screening hundreds of clones [11]. This strain presented altered cell morphology and a severe loss of viability during stationary phase, and these changes were not observed for the *cspD* mutant, indicating that the CspC has a specific role in cell viability [11].

Several important changes occur at the onset of stationary phase, mainly diminishing the overall metabolism and triggering the expression of specific genes to adapt the cells to withstand nutrient scarcity [16]. Moreover, adjusting the respiratory rates and enhancing stress response mechanisms are equally important to cope with the oxidative stress that is increased at this stage [16]. As an oligotrophic bacterium, *C. crescentus* displays nutritional versatility, being capable of growing in several distinct carbon sources besides sugars, such as aromatic compounds [17], fatty acids [18] and acetate [19].

In this work, we address the role of CspC at stationary phase using a global transcriptional profiling experiment. The results indicate that CspC is necessary for the expression of several genes involved in adaptation to nutritional starvation, particularly of the genes encoding enzymes of the glyoxylate cycle, and that the mutant *cspC* strain is unable to grow in acetate as the sole carbon source. This effect was at least partially due to post-transcriptional regulation of the mRNA encoding one key enzyme of the pathway, with CspC increasing the stability of this mRNA.

## Methods

### Bacterial strains and growth conditions

The strains utilized were derived from *Caulobacter crescentus* synchronizable strain NA1000 [20]. None of the bacterial strains used in this study required ethical approval to use. NA1000 and *cspC* (Δ*cspC*::ΩSpec^R^) [11] strains were cultured aerobically in peptone-yeast extract (PYE) medium or M2 minimal medium at 30 °C [21] supplemented with tetracycline (1 μg ml^−1^) as necessary. M2 containing 0.2 % acetate as the sole carbon source (M2A) was used in specific growth assays. Cultures of NA1000 and *cspC* mutant strains were grown up to midlog phase (Optical Density (OD_600_) = 0.3), the cells were pelleted and suspended in M2A. Growth rates were determined by measuring the OD_600_ at regular intervals. Aliquots were centrifuged at 12,000 × g and 4 °C for 5 min, and the supernatants were stored at −20 °C until further analysis of the consumption of acetate. Total acetate from the medium was determined by high-performance liquid chromatography (HPLC). A volume of 10 μl was injected into a Dionex HPLC (Ultimate 3000, Thermo Fisher Scientific Inc, Waltham, MA, USA) using a cation-exchange column (Aminex-HPX-87H). The system was equipped with differential refractometer (Shodex IR-101). Mobile phase was 5 mM H_2_SO_4_ at a flow rate of 0.6 ml/min.

### Expression assays

The expression the *cspC* was measured during nutritional starvation for glucose or nitrogen. For the glucose depletion assays, NA1000 cultures containing a transcriptional *cspC-lacZ* fusion cloned into pRK*lacZ*290 [22] were diluted to OD_600_ = 0.05 and at midlog phase (OD_600_ = 0.3) were centrifuged and resuspended in either M2 medium (control) or M2 medium lacking glucose or lacking NH_4_Cl. A sample of each culture was taken for expression assays and the cultures were further incubated for 3 h, when a second sample was taken. Expression was determined by β-galactosidase activity assays [23]. The assessment of *cspC* expression in other media was performed by diluting cultures of NA1000 harboring the *cspC-lacZ* fusion to an OD_600_ = 0.1 in the following media: M2G (complete M2 medium), M2P (M2 with 0.2 % peptone instead of glucose), PYE and PYEG (PYE plus 0.2 % glucose). Expression was assessed at different time points by β-galactosidase activity assay [23]. *sigU* and *sigT* transcriptional fusions were kindly provided by Suely Lopes Gomes [24]. β-galactosidase activity from these strains was determined at midlog phase (OD_600_ = 0.5) and after 24 h growth (OD_600_ = 1.2–1.3) [23] Statistical significance of the results was assessed using one-way analysis of variance (ANOVA) followed by Turkey-Kramer comparison test as necessary.

### DNA microarray analysis

DNA microarray global transcriptional profiling experiments were carried out using RNA isolated from NA1000 and *cspC* strains grown in PYE up to mid-exponential phase (OD_600_ = 0.4–0.5) and stationary phase (24 h, OD_600_ = 1.2–1.3). The *cspC* mutant strain presents a growth rate in PYE medium that is close to WT. Growth of the cultures for RNA isolation was monitored by absorbance readings, and after 24 h both cultures were well into stationary phase.

Slides containing tiled 50mer probes corresponding to all 3767 predicted open reading frames (ORFs) in the genome of *C. crescentus* CB15 were used (Agilent Technologies). The set of probes covered the region around the start codon (−300 to +200 relative to the translation start site) of each predicted ORF, so that the last four probes corresponding to each gene most likely comprise its transcribed region; since only coding regions were of our interest in this work, we considered only the four last oligonucleotides of a given ORF. Total RNA from independent cultures was isolated for each strain with Trizol reagent (Invitrogen) following the manufacturer’s instructions. RNA was quantified with NanoDrop 2000 (Thermo Scientific) followed by treatment with DNAse I (Fermentas). cDNA was generated with the FairPlay III Microarray Labeling Kit (Agilent Technologies), and labeled with Alexa 555 (Cy3) or Alexa Fluor 647 (Cy5) mono-reactive fluorescent dyes. Hybridization was carried out for 24 h at 65 °C and 10 rpm and arrays were scanned for the Cy3 and Cy5 fluorescent signals using the SureScan Microarray Scanner (Agilent Technologies). Data extraction and normalization was performed with the Feature Extraction Software 9.0 (Agilent Technologies). A gene was considered as upregulated or downregulated if it showed a minimum of 2.5 fold change relative to the control in at least three out of the four last oligonucleotides in all biological replicates. The DNA microarray experiments were carried out from three biological replicates for the *cspC* x NA1000 analysis, and two biological replicates for the NA1000 exponential phase x stationary phase analysis.

### RNA isolation and quantitative real-time RT-PCR

RNA was isolated with TRIzol reagent (Invitrogen) according to the manufacturer’s instructions. The synthesis of cDNA was carried out from 3 μg RNA, which was previously treated with RNase-free DNase I (Fermentas). Reverse transcription was performed using Super Script II (Invitrogen) and random hexamers according to the manufacturer’s instructions of “SuperScript III First Strand Synthesis for RT-PCR Kit” (Invitrogen). Specific primers for each gene were used [see Additional file 1: Table S1] for the amplification with Maxima SYBR Green/ROX qPCR Master Mix (Fermentas), and the reactions were carried out in the ABI 7500 real-time PCR system (Life Technologies). The gene chosen as endogenous control for normalizing the results was CC0088, which showed no variation in expression in the DNA microarray experiments. The primers were confirmed to be equally efficient for amplification in the conditions tested, so the method of 2^-∆∆CT^ [25] was used for calculating the relative gene expression.

### mRNA decay assays

In order to determine the decay rate of *aceA* and *aceB* mRNAs, overnight cultures of NA1000 (pMR20), *cspC* (pMR20) and complemented strain *cspC* (pMR20-*cspC*) [11] were diluted to an OD_600_ = 0.1 in 50 ml PYE medium. Cells were grown up to stationary phase (24 h), and transcription was interrupted by rifampin (20 μg/ml) addition. Five aliquots were taken from each culture at the indicated time points. Total RNA was extracted and treated as described above, and used in real time quantitative RT-PCR reactions with the appropriate primers [see Additional file 1: Figure S1]. The *C*_*T*_ obtained for 0 h-time point was used as a reference to calculate the relative mRNA levels in samples removed at time points after rifampin addition.

## Results

### *cspC* is induced in medium-dependent conditions

**Fig. 1:**
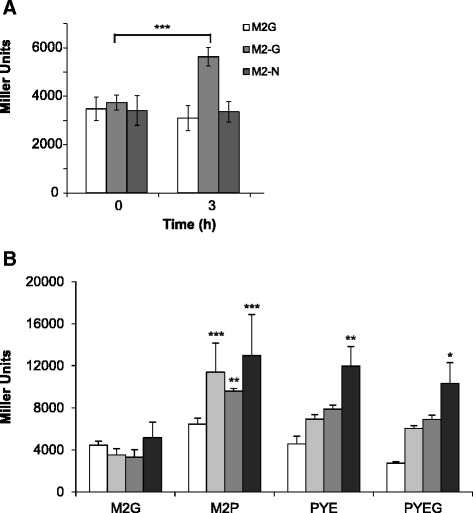
*cspC* expression under different media conditions. Cultures of NA1000 harboring the *cspC-lacZ* transcriptional fusion were grown in the indicated media, and *cspC* expression was assessed by β-galactosidase activity assays. **a** *cspC* expression under nutrient starvation. Cultures grown in M2G to an OD_600_ = 0.3 were washed and ressuspended in M2G (control), M2-G (without glucose) or M2–N (without ammonium chloride). **b** *cspC* expression in different types of media. Cultures were grown in the following media: M2 with 0.2 % glucose (M2G); M2 in which glucose was replaced by 0.2 % peptone (M2P); PYE and PYE containing 0.2 % glucose (PYEG). Beta-galactosidase activity assays were performed at time points: 0 h, 18 h, 24 h, and 48 h. Asterisks indicate a statistically significant difference in a two-way ANOVA test, with *p* < 0.05 (*), *p* < 0.01 (**) or *p* < 0.001 (***). Statistical analysis of data indicated in (B) is relative to the same time point in M2G. Data are mean values of at least two independent experiments. Vertical black bars indicate the standard deviation

The *cspC* gene was described as essential for *C. crescentus* growth [15], and the *cspC* mutant shows a severe decrease in viability at stationary phase [11]. The CspC protein accumulates during early stationary phase, indicating that the nutritional condition of the medium could affect its expression [11]. To investigate this matter, the expression driven from the *cspC* promoter in a *cspC-lacZ* fusion was measured by β-galactosidase activity assays. NA1000 cells carrying *cspC-lacZ* construct were grown in complete M2 medium (M2G), washed and resuspended in M2G or M2 lacking either glucose or ammonium acetate (Fig. 1a). The results showed that the expression of *cspC* increased significantly in the absence of glucose, but not ammonium, indicating that carbon starvation might be a signal for the expression of this gene. Therefore, we speculated whether its essentiality for stationary phase survival might be related to adaptation to a condition where there is a shortage of available carbon sources. The same assay was carried out in different nutrient media, in order to determine if the type of carbon source present would interfere with *cspC* expression. As observed previously for the CspC protein [11], *cspC* gene expression in rich PYE medium increased at least two fold after 48 h incubation (Fig. 1b). However, the same was not true when cells were grown in defined M2G medium, indicating that some medium components were affecting its expression. The first obvious difference is that in PYE the carbon and nitrogen sources are provided by amino acids, whereas in M2G carbon source is glucose and nitrogen source is ammonium chloride. Cells were then grown in M2 medium lacking glucose and supplied with peptone, and *cspC* was highly induced after 18 h of growth (M2P in Fig. 1b). The results indicate that *cspC* is induced when the cells enter stationary phase maybe as a result of the available amino acids becoming in short supply. The differences observed in *cspC* expression were probably not due to distinct growth rates, since the addition of glucose to PYE, which increases growth, did not have an effect in expression (PYEG in Fig. 1b).

### Global transcriptomic analyses of the *cspC* mutant

Our hypothesis was that as a nucleic acid binding protein, CspC could modulate gene expression to help adapting cells in response to carbon starvation. In order to address this question, the approach was to establish whether the lack of *cspC* causes a change in global gene expression. DNA microarray transcriptional profiling experiments were carried out comparing *cspC* and the parental NA1000 strain at mid-exponential phase (Tables 1 and 2) and after cells entered stationary phase in PYE medium (Tables 3 and 4). In order to identify those genes that had expression affected in the *cspC* mutant and were also induced at stationary phase, a third DNA microarray analysis was performed comparing gene expression between exponential and stationary phases (Tables 3 and 4, fifth columns).

**Table 1:** Genes downregulated in the *cspC* mutant at exponential phase

Gene CB15	Gene NA1000	Predicted function	Fold change
			(∆*cspC*/WT)^a^
Respiratory Chain			
CC_0763	CCNA_00801	cytochrome bd-type quinol oxidase, subunit II	0.38
Toxin-antitoxin system			
CC_0873	CCNA_00916	toxin protein parE-1	0.25
Carbohydrate Metabolism			
CC_3655	CCNA_03770	malate dehydrogenase	0.22
Hypothetical			
CC_2280	CCNA_02363	Hypothetical protein	0.33
CC_2645	CCNA_02728	Hypothetical protein	0.32
CC_3115	CCNA_03214	Hypothetical protein	0.26

^a^Values are fold changes in the expression levels comparing the null *cspC* mutant (∆*cspC*) with wild type cells (WT). The values are the means of three biological replicates

**Table 2:** Genes upregulated in the *cspC* mutant at exponential phase

Gene CB15	Gene NA1000	Predicted function	Fold change
			(∆*cspC*/WT)^a^
Amino acid metabolism			
CC_1382	CCNA_01446	LL-diaminopimelate aminotransferase	4.37
CC_1397	CCNA_01463	3-deoxy-7-phosphoheptulonate synthase	10.70
Carbohydrate metabolism			
CC_1396	CCNA_01462	lactate 2-monooxygenase	7.80
Protein synthesis			
CC_1870	CCNA_01946	tyrosyl-tRNA synthetase	9.85
Oxydative stress response			
CC_1871	CCNA_01947	thioredoxin peroxidase	3.99

^a^Values are fold changes in the expression levels comparing the null *cspC* mutant (∆*cspC*) with wild type cells (WT). The values are the means of three biological replicates

**Table 3:** Genes downregulated in the *cspC* mutant at stationary phase

Gene CB15	Gene NA1000	Predicted function	Fold change (∆*cspC/*WT)^a^	Fold change (WTstat/WTexp)^b^	Upregulated in carbon limitation^c^
Carbohydrate Metabolism					
CC_1764	CCNA_01841	isocitrate lyase	0.11	71.6^d^	Yes
CC_1765	CCNA_01843	malate synthase	0.10	88.7^d^	Yes
CC_3655	CCNA_03770	malate dehydrogenase	0.06	2.1	No
CC_3580	CCNA_03695	aldehyde dehydrogenase	0.25	82.4^d^	Yes
CC_3581	CCNA_03696	acetyl-CoA synthetase	0.25	20.0^d^	No
Amino acid Metabolism					
CC_0167	CCNA_00166	putative transglutaminase-like cysteine proteinase	0.20	2.2	Yes
CC_2533	CCNA_02616	4-hydroxyphenylpyruvate dioxygenase	0.36	1.2	No
Transport					
CC_0579	CCNA_00615	TonB-dependent receptor	0.22	8.1^d^	No
Motility					
CC_0793	CCNA_00835	flagellin FljN	0.17	0.7	No
CC_1460	CCNA_01527	flagellin FljL	0.33	0.2^d^	No
CC_1461	CCNA_01528	flagellin FljK	0.28	0.9	Yes
CC_2948	CCNA_03043	type IV pilin protein PilA	0.25	0.8	No
CC_3025	CCNA_03120	chemotaxis protein CheW	0.14	1.4	Yes
Oxidative stress response					
CC_1777	CCNA_01855	iron/manganese superoxide dismutase	0.35	1.3	No
Toxin-antitoxin systems					
CC_2513	CCNA_02598	toxin protein relE_2_	0.39	10.1^d^	Yes
CC_2880	CCNA_02974	toxin protein relE_3_	0.39	14.1^d^	No
Signal Transduction and Transcription					
CC_0903	CCNA_00948	CtrA inhibitory protein SciP	0.31	1.0	Yes
Proteins with no assigned category					
CC_0451	CCNA_00483	Zn-dependent hydrolase, glyoxalase II family	0.33	0.5	No
CC_0557	CCNA_00592	ferritin superfamily protein	0.34	2.7	Yes
CC_0679	CCNA_00718	abortive infection protein	0.30	2.1	Yes
CC_0707	CCNA_00744	GIY-YIG endonuclease domain protein	0.28	4.5^d^	Yes
CC_1343	CCNA_01405	GIY-YIG domain protein	0.32	16.9^d^	Yes
CC_2696	CCNA_02779	LysM family peptidoglycan binding protein	0.35	6.6^d^	No
CC_2869	CCNA_02962	HicB-family RNase H fold protein	0.30	11.9^d^	Yes
CC_3153	CCNA_03255	PemK-family protein	0.34	4.5^d^	Yes
CC_3582	CCNA_03697	ligand-binding SRPBCC domain protein	0.25	14.5^d^	No
Hypothetical					
CC_0559	CCNA_00594	Hypothetical protein	0.14	10.6^d^	Yes
CC_0681	Unannotated		0.05	4.4^d^	No
CC_0682	Unannotated		0.19	6.4^d^	No
CC_0781	CCNA_00822	Hypothetical protein	0.10	13.2^d^	Yes
CC_0997	Unannotated		0.31	22.5^d^	No
CC_1392	CCNA_01458	Hypothetical protein	0.34	9.3^d^	No
CC_2184	Unannotated		0.18	12.4^d^	Yes
CC_2348	CCNA_02433	Hypothetical protein	0.26	6.6^d^	Yes
CC_2645	CCNA_02728	Hypothetical protein	0.26	38.1^d^	Yes
CC_3207	CCNA_03313	Hypothetical protein	0.33	38.7^d^	Yes
CC_3654	CCNA_03769	Hypothetical protein	0.23	5.8^d^	No

^a^Values are fold changes in the expression levels comparing the null *cspC* mutant (∆*cspC*) with wild type cells (WT). The values are the means of three biological replicates

^b^Values are fold changes in the expression levels comparing wild type cells at stationary phase (WT stat) versus exponential phase (WT exp). The values are the means of two biological replicates

^c^Genes upregulated in carbon limitation in previous work [26]

^d^Genes significantly up- or downregulated at stationary phase with respect to exponential phase

**Table 4:** Genes upregulated in the *cspC* mutant at stationary phase

Gene CB15	Gene NA1000	Predicted function	Fold change (∆*cspC*/WT)^a^	Fold change (WTstat/WTexp)^b^	Downregulated in carbon limitation^c^
Sulfur Metabolism					
CC_0286	CCNA_00288	ABC-type sulfate transport system, periplasmic component	4.43	2.7^d^	Yes
CC_1119	CCNA_01177	sulfite reductase, beta subunit	5.22	2.0	Yes
CC_1482	CCNA_01549	sulfate adenylyltransferase subunit 1/ /adenylylsulfate kinase	8.94	0.9	Yes
CC_1483	CCNA_01550	sulfate adenylyltransferase, subunit 2	5.36	2.1	Yes
Transport					
CC_1666	CCNA_01738	TonB-dependent receptor	5.41	3.2^d^	No
CC_1991	CCNA_02070	protein translocase subnit SecD	2.62	0.2^d^	Yes
CC_2664	CCNA_02747	ABC transporter substrate-binding protein	6.75	1.1	Yes
Respiratory Chain					
CC_1401	CCNA_01467	cytochrome cbb_3_ oxidase subunit I CcoN	3.25	1.8	No
CC_1402	CCNA_01468	cytochrome cbb_3_ oxidase, cytochrome c subunit CcoO	3.91	2.2	No
CC_1403	CCNA_01469	cytochrome cbb_3_ oxidase, subunit CcoQ	3.00	2.2	No
Amino Acid Metabolism					
CC_0050	CCNA_00048	S-adenosylmethionine synthetase	3.57	0.5	No
CC_0482	CCNA_00515	5-methyltetrahydropteroyltriglutamate--homocysteine methyltransferase	5.57	0.7	Yes
CC_0581	CCNA_00617	arginine N-succinyltransferase beta chain	4.63	4.8^d^	No
CC_1397	CCNA_01463	3-deoxy-7-phosphoheptulonate synthase	3.65	3.2^d^	No
Signal Transduction					
CC_0285	CCNA_00287	photosensory histidine protein kinase LovK	4.44	1.7	No
Protein Synthesis					
CC_0464	CCNA_00496	threonyl -tRNA synthetase	4.91	0.5	Yes
CC_1870	CCNA_01946	tyrosyl-tRNA synthetase	3.09	1.1	No
Proteases					
CC_0878	CCNA_00922	ATP-dependent Clp protease. ATP-binding subunit ClpB	3.14	1.5	No
CC_1960	CCNA_02037	ATP-dependent endopeptidase Lon	2.93	1.8	No
Cofactors metabolism					
CC_2912	CCNA_03006	quinolinate synthetase A	4.75	0.9	No
Hypothetical					
CC_0287	CCNA_00289	hypothetical protein	5.62	1.7	Yes
CC_2624	Unannotated	hypothetical protein	2.94	2.8	No
CC_2699	CCNA_02782	hypothetical protein	4.41	2.0	No

^a^Values are fold changes in the expression levels comparing the null *cspC* mutant (∆*cspC*) with wild type cells (WT). The values are the means of three biological replicates

^b^Values are fold changes in the expression levels comparing wild type cells at stationary phase (WT stat) versus exponential phase (WT exp). The values are the means of two biological replicates

^c^Genes downregulated in carbon limitation in previous work [26]

^d^Genes significantly up- or downregulated at stationary phase with respect to exponential phase

Table 1 shows genes downregulated in the mutant at exponential phase compared to the wild type, grouped by functional categories. Very few genes showed differential expression at this growth phase, being 6 genes downregulated (Table 1) and five genes upregulated (Table 2). Among the downregulated genes are those encoding subunit II of cytochrome bd-type quinol oxidase, malate dehydrogenase *(mdh*), and a gene encoding the toxin parE-1, besides three hypothetical proteins. The genes upregulated in the *cspC* mutant in exponential phase are functionally involved in amino acid synthesis, specifically, lysine, phenylalanine, tyrosine, and tryptophan, as reflected by LL-diaminopimelate aminotransferase (CC_1382) and 3-deoxy-7-phosphoheptulonate synthase (CC_1397). The *mdh* and CC2645 genes were also downregulated in stationary phase and CC1871 was also upregulated in stationary phase (Tables 3 and 4).

**Fig. 2:**
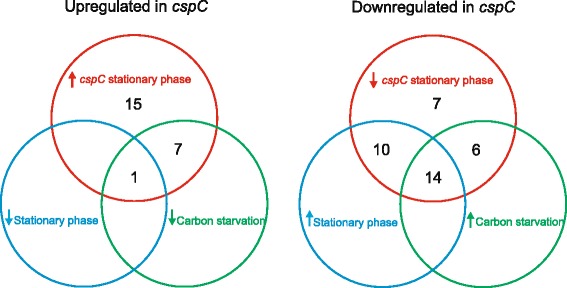
Genes differentially expressed in the *cspC* mutant identified by microarray experiments. The Venn diagrams highlight the genes differentially expressed in three microarray experiments: *cspC* mutant x wild type at stationary phase exponential x stationary phase, and carbon starvation [26]. The diagrams show the number of genes upregulated in the *cspC* mutant and downregulated in the other two conditions, and the genes downregulated in the *cspC* mutant and upregulated in the other conditions

**Fig. 3:**
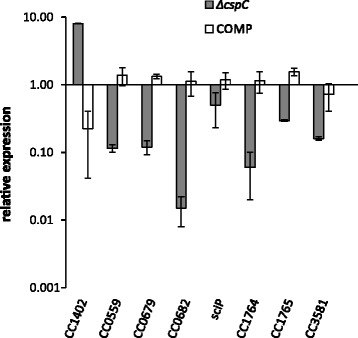
Expression analyses of selected genes in *C. crescentus cspC* or complemented strains. qRT-PCR experiments were performed with total RNA extracted from stationary phase cultures. Results were normalized using gene CC0088 as the endogenous control, which was constitutively expressed in the samples analyzed. The expression values for NA1000 were used as reference for each gene. Data are mean values of two independent experiments; bars represent the standard error. Strains are noted as follows: Comp, complemented strain (*cspC* (pMR20-*cspC))* and ∆*cspC,* ∆*cspC::*ΩSpec^R^ mutant strain

Since *cspC* is induced at stationary phase, we expected a higher impact of the lack of this gene at this growth phase. The results showed that the absence of *cspC* affected the transcription of 60 genes at stationary phase, fifteen of which were substantially affected (>5 fold) compared to the wild type strain (Table 3 and 4). Of the 37 genes downregulated in the *cspC* mutant, 24 were more expressed at stationary phase (as determined in the exponential x stationary phase microarray experiments), 20 were more expressed in carbon starvation [26] and 14 were differentially expressed in all three conditions (Fig. 2). Of the 23 genes upregulated in the *cspC* mutant, 8 were more expressed in carbon starvation [26] and only one was differentially expressed in all three conditions (Fig. 2). The results show a good correlation between genes downregulated in the *cspC* mutant with induction at stationary phase and by carbon starvation, but the upregulated genes do not show this profile. To validate the microarray analyses, we performed quantitative real time RT-PCR of eight selected genes whose expression levels were significantly altered in the microarray experiments at stationary phase (Fig. 3). Gene expression was determined both in the *cspC* mutant and in the complemented strain, and the results confirmed that CspC is necessary for the normal level of expression of these genes at this phase.

The most prominent groups of downregulated genes in the *cspC* mutant at stationary phase are involved in amino acid and carbohydrate metabolism, chemotaxis and motility, signal transduction and transcription, and many genes encode hypothetical proteins (Table 3). Comparing our microarray data with the large-scale transcriptomic study performed on carbon starvation in *C. crescentus* [26], we observed that six out of the eleven genes encoding hypothetical proteins identified as downregulated in the *cspC* mutant are also induced by carbon limitation. Of these, two genes (CC3207 and CC2348) were induced more than twentyfold when the cells were under carbon starvation [26].

**Fig. 4:**
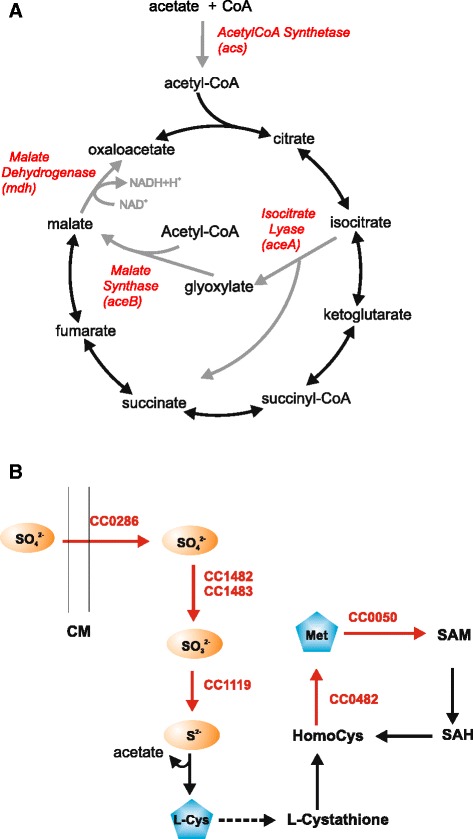
Metabolic pathways affected in the *cspC* mutant. **a** Schematic representation of the Glyoxylate Cycle, indicating genes that were downregulated in the *cspC* mutant and their respective products (in red). Metabolic conversions predicted to be diminished in the mutant are shown by gray arrows. **b** Schematic representation of the assimilatory sulfate reduction and methionine biosynthesis pathways. Enzymes whose coding genes were upregulated in the *cspC* mutant are indicated in red. CM, cytoplasmic membrane; SAM, S-adenosyl methionine; SAH, S-adenosyl homocysteine

An operon comprising two downregulated genes in the *cspC* mutant at stationary phase encodes the enzymes aldehyde dehydrogenase (*aldB*) and acetyl-CoA synthetase, which convert the acetaldehyde generated by the metabolism of alternative carbon sources into acetyl-CoA. Three other genes important for acetyl-CoA metabolism, *aceA*, *aceB* and *mdh*, are also severely downregulated in the *cspC* mutant (Fig. 4a), and will be discussed in more detail below. A gene related to oxidative stress, encoding an iron/manganese superoxide dismutase (CC1777) was downregulated in the *cspC* mutant. Two genes encoding toxins, RelE_2_ (CC2513) and RelE_3_ (CC2880) were also downregulated, and it has been reported [27] that the expression of the *relBE*_*3*_ operon increased in response to oxidative stress.

Importantly, the *sciP* gene, encoding an essential transcription factor involved in repressing *ctrA* and CtrA target genes [28] showed a 3.6-fold decrease in expression in the *cspC* mutant at stationary phase. CtrA is a global transcriptional regulator that control approximately 100 cell cycle-regulated genes, including many flagellar and chemotaxis genes [29]. However, only a few genes involved in flagellum assembly (CC0793, CC1460-01, CC2948) and chemotaxis (CC3025) were downregulated in the *cspC* mutant. Three of these genes (CC3025, CC146) were also induced by carbon limitation [26].

The genes upregulated in *cspC* at stationary phase participate in different metabolic pathways, such as amino acid and sulfur metabolism, transport, protein synthesis and energy metabolism (Table 4). Three respiration-related genes encoding subunits of *cbb*_*3*_ oxidase of the electron transport chain (CC1401-03) showed significant increase in expression in the *cspC* mutant. In *Rhodobacter sphaeroides*, the *cbb*_*3*_ terminal oxidase was associated with the redox-responsive control of PrrB activity [30].

Interestingly, genes encoding components of the sulfate assimilation pathway were more expressed in the *cspC* mutant (Fig. 4b). These include the enzymes that incorporate sulfate-derived sulfur into cysteine: sulfate adenylate transferase/adenylylsulfate kinase (CC1482-1483), a periplasmic sulfate-binding protein that possibly delivers sulfate to the ABC transport system (CC0286), and a sulfite reductase (CC1119). Two genes encoding enzymes of methionine biosynthesis pathways were also upregulated: adenosylmethionine synthetase (CC0050) and S-adenosyl-L-homocysteine hydrolase (CC0482). All the genes above were downregulated in condition of carbon starvation [26].

**Fig. 5:**
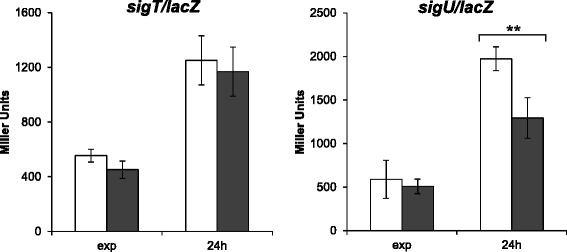
Expression of *sigU* and *sigT*. β-galactosidase activity assays of wild type and *cspC* strains carrying either a *sigU* (**a**) or *sigT* (**b**) transcriptional fusion to *lacZ* was measured at exponential-phase and 24 h growth. The results are the average of three independent assays; bars represent the standard error. Significance was assessed using 1-way ANOVA and Tukey post-test; *p* < 0.01 (**)

The mRNA levels of the histidine kinase LovK increased in the *cspC* mutant. The histidine kinase LovK together with its cognate response regulator LovR, comprise a two-component system functioning as a negative regulator of the *C. crescentus* general stress pathway [31]. LovK-LovR regulates this pathway by controlling the phosphorylation state of PhyR, leading to activation of σ^T^, which in turn transcribes σ^U^ [31, 32]. Although *sigU* was shown to be less expressed in two out of three of the microarray experiments, it did not fit the cutoff criteria used to be considered as differentially expressed. In order to confirm this result β-galactosidase activity assays (Fig. 5) were carried out, and confirmed that *sigU* is less expressed in the *cspC* mutant. On the other hand, *sigT* did not show differential expression, both in the DNA microarrays and β-galactosidase activity assays (Fig. 5).

### *aceA* mRNA is less stable in the *cspC* strain

Interestingly, our array data showed that genes encoding two key enzymes of the glyoxylate cycle, isocitrate lyase (*aceA*) and malate synthase (*aceB*), and two other enzymes related to the same pathway, malate dehydrogenase (*mdh*) and acetyl-CoA synthetase (*acs*), were severely downregulated in the *cspC* mutant at stationary phase (Fig. 4a). Three of these *cspC*-dependent genes are highly induced upon entry into stationary phase, as determined by DNA microarray assays: *aceA,* 71.6-fold; *aceB,* 88.7-fold; and *acs*, 20-fold (Table 3), indicating that the glyoxylate cycle is important at this stage. In *C. crescentus* NA1000, *aceA* is in an operon with a downstream ORF (CCNA_01842) encoding a hypothetical protein, but these two genes are not co-transcribed with the downstream gene *aceB* [33].

**Fig. 6:**
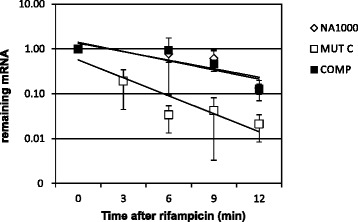
mRNA decay assays of *aceA* mRNA in *cspC* strain at stationary phase. mRNA levels were measured as a function of time after rifampicin treatment using qRT-PCR. mRNA levels quantified by qRT-PCR were fit to a single exponential decay curve to measure the mRNA half-life. The half-lives shown are representative of those from two independent rifampin chase experiments with comparable results. Strains are labeled as indicated: open rhombuses, wild type; filled squares, complemented strain *cspC* (pMR20-*cspC*) and open squares, *cspC* mutant strain

To determine whether CspC could affect stability of the mRNAs of the glyoxylate cycle genes, the rate of decay of *aceA* mRNA was determined in wild type, *cspC* mutant and complemented strains at stationary phase (Fig. 6). The mRNA decay assays revealed that *aceA* mRNA decays more rapidly in the *cspC* strain than in the parental and complemented strains. One hypothesis could be that CspC may bind to *aceA* mRNA stabilizing it, probably by preventing access of RNAses, but we cannot exclude the possibility that this effect could be indirect, for example mediated by a small regulatory RNA. We have performed the same analysis for *aceB*, but the results did not show a more pronounced rate of decay in the *cspC* mutant (data not shown), indicating that its lower mRNA levels may be the result of a different mechanism. On the other hand, if the effect on *aceB* stability was very minor, it could have been below our detection with the methods and conditions used.

### CspC is required for bacterial growth in acetate

**Fig. 7:**
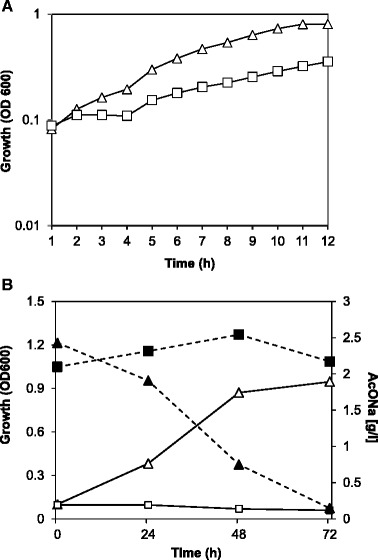
Growth and acetate consumption of the NA1000 and *cspC* strains. Cultures of the wild type (white triangles) and the *cspC* mutant (white squares) grown on M2G (0.2 % glucose) were washed with M2 salts and inoculated into fresh M2G medium (**a**) or M2 where glucose was substituted by 0.2 % sodium acetate (**b**). Cell growth was monitored by measuring the OD_600_. Acetate consumption (NA1000: filled triangles; *cspC*: filled squares) was determined at each time point.

To confirm the requirement of *cspC* for growth in acetate as unique carbon source, the *cspC* and wild-type strains were grown in minimal M2 medium containing acetate as the sole carbon source. Growth was determined during five days and the consumption of acetate was quantified (Fig. 7). Although the NA1000 strain presented a long growth delay, with a lag phase of 48 h, this strain was able to reach stationary phase and entirely consumed the acetate present in medium. In contrast, neither growth nor acetate consumption was detectable for the *cspC* mutant after five days, indicating that this strain is unable to grow under these conditions.

## Discussion

The *cspC* mutant shows a severe loss of viability during stationary phase, indicating that the CspC has a main role in adaptation to this phase [11]. In the present work, we have analyzed the effect of the deletion of *cspC* in *C. crescentus* global gene expression both at exponential and stationary phases. The number of genes that were differentially expressed in exponential phase was much smaller than at stationary phase, confirming that CspC has a predominant role at this latter phase. The most interesting trend observed at exponential phase was that many upregulated genes are involved with amino acid synthesis, suggesting that the mutant cell is sensing a situation of amino acid starvation, even in a growth medium with sufficient amino acids.

Among the downregulated genes in the *cspC* strain at stationary phase, the *sciP* gene was also characterized as an essential gene [28]. This transcription factor represses transcription of late predivisional cell and swarmer cell specific genes that have been activated by CtrA and also represses CtrA; moreover, downregulation of CtrA has been observed at stationary phase [34]. It is reasonable to suppose that SciP could also repress CtrA also during stationary phase. However, *ctrA* mRNA levels were not affected in the *cspC* microarray experiments, and only three genes (*pilA*, *fliL* and CC0681) belonging to the SciP regulon [28] were downregulated in the *cspC* mutant relative to the wild-type. *fliL* and CC0681 are not members of the CtrA regulon but their promoters contain a SciP DNA binding motif suggesting that SciP participate directly in their regulation.

Interestingly, the expression levels of the heat shock genes *clpB* and *lon* were increased in the *cspC* mutant at stationary phase. Lon and ClpB are involved in refolding or degradation of misfolded proteins and disaggregating insoluble protein aggregates, respectively [35, 36], so the increase in the expression of these genes suggests that misfolded proteins might be accumulating in the mutant strain, as a sign of stress. SciP was described as a Lon substrate in *C. crescentus* [37], so low expression of SciP could be occurring both at the mRNA and protein level.

The positive regulation of the general stress response include the ECF sigma factor σ^T^, its anti-σ factor NepR, the anti-anti-σ factor PhyR, and the transmembrane sensor kinase PhyK [31, 32]. *sigU* expression was diminished in the *cspC* mutant; however, other genes that are SigT-dependent [24] did not show altered expression, suggesting that the CspC effect on SigU expression might be post-transcriptional. The absence of correspondence between the SigT and CspC regulons indicates that *sigU* expression is affected by CspC in a SigT-independent manner. Independent regulation between SigT and SigU has been previously reported in ∆*fixJ* e ∆*ftrA* strains, where *sigU* expression was altered but the expression of *sigT* was not modified [38], indicating that *sigU* is subject to a second level of regulation.

Among the genes strongly downregulated there is a gene encoding a 1.1 kb non-coding RNA between ORF CC0680 and CC0683. The existence of this long RNA was predicted in earlier work [39] and confirmed recently [33]. This transcript was identified as part of the SciP regulon [28], and a recent study from our group showed that its expression is also regulated by Fur [40]. *cspC* expression does not respond to the change in iron levels and is not regulated by Fur (data not shown), so the decrease of the levels of this transcript could be via a decrease in its stabilization.

The decreased flux through the electron transport chain at stationary phase may be a defense mechanism to protect against reactive oxygen species (ROS) generated during electron transport, as well as prevent an over consumption of endogenous reserves [16]. The upregulation of several genes indicate that the *cspC* mutant could be under significant oxidative stress at stationary phase. The genes encoding cytochromes of the *cbb*_*3*_ complex (CC1401-03) were upregulated in the *cspC* mutant. The *cbb*_*3*_ respiratory complex has high affinity for oxygen, and besides its major role in respiration under conditions of low oxygen pressure, it has been implicated in signal transduction in *Rhodobacter sphaeroides* [41]. These authors have proposed that *cbb*_*3*_ oxidase acts as an O_2_ sensor, generating an inhibitory signal to repress photosynthesis gene expression via the two component system PrrBA. Other terminal oxidases have been implicated in responding to oxidative stress [42]. The increased expression of LovK, that was described as a redox sensor in the cytoplasm and a light sensor in *C. crescentus* [43] and of the superoxide dismutase SodA reinforce this hypothesis.

The glyoxylate cycle is an anapleurotic pathway of the tricarboxylic acid cycle that allows growth on substrates that are converted into acetyl-CoA, e.g. fatty acids, acetate or ethanol [44]. The repression of the glyoxylate cycle genes in the mutant, preventing the conversion of acetyl-CoA into oxaloacetate, may impair growth from other carbon sources, such as fatty acids. In fact, the *cspC* mutant is unable to grow in acetate as the sole carbon source (Fig. 7). The remarkable induction of the genes of sulfur metabolism in the *cspC* mutant indicates that the cells are actively increasing the input of sulfate and that it is probably being funneled to the methionine biosynthesis pathway. Previous work has shown that this effect is a result of the methionine pool becomes limiting for growth in acetate-treated cells, and growth arrest may be reversed by the addition of exogenous methionine [45]. Moreover, *E. coli cspA* has been shown to be induced by the addition of homocysteine to the medium, and this induction is likely to be caused by the depletion of isoleucine that arises in treated cells [46]. The authors proposed that ribosomal stalling caused by amino acid depletion acts as the signal leading to induction of *cspA*. In *C. crescentus*, stationary phase caused by the shortage of amino acid supply may lead to the same condition, triggering the expression of *cspC*.

The similarities found here between the three microarrays data (carbon starvation, exponential x stationary phase, and *cspC* mutant x wild type at stationary phase) strongly suggests that *cspC* has an important role in nutrient starvation response at this phase. The modulation of *aceA* mRNA levels by CspC has unveiled a molecular mechanism that may be relevant in other bacteria. In *Corynebacterium glutamicum* it was shown that the glyoxylate cycle is regulated at the mRNA level via dowregulation of *aceA* by RNase E/G, mediated by the secondary structure of the 3’ UTR of *aceA* mRNA [47]. The analysis of the predicted 3’-UTR of *C. crescentus aceA*-CCNA01842 mRNA showed a putative GC-rich hairpin loop, but there is no predicted Rho-independent transcription terminators. Interestingly, we have identified a secondary structure similar to the one in *C. glutamicum aceA* at the 3’ UTR in region of *C. crescentus aceB* mRNA, but the involvement of RNase E in its degradation is still to be determined. It has been proposed for *E. coli* that the long-chain fatty acids from the cytoplasmic membrane may provide a source of carbon and energy for starved cells [16]. This impairment of the glyoxylate cycle could be one of the reasons for the loss of viability of the *cspC* mutant at stationary phase, when there is the need for the metabolism of alternative carbon sources. The conditions at stationary growth phase are comparable to those in the scarce natural environment in which bacteria can be found. One plausible explanation for the activation of glyoxylate cycle at stationary phase is that *C. crescentus* could utilize acetate or other acids as alternative carbon sources derived from fermentation product of other bacteria that live in the same environment.

## Conclusions

The stationary phase-induced RNA binding protein CspC has an important role in gene expression at this phase. Several genes have their expression levels downregulated by the absence of CspC, including the essential gene *sciP*, the ECF sigma factor *sigU*, as well as the genes for enzymes of the glyoxylate cycle and for oxidative stress response. On the other hand, genes for reductive sulfate assimilation and methionine biosynthesis are upregulated, probably as a response to the inefficiency of the glyoxylate cycle. At least in the case of *aceA*, the downregulation may be attributed to the shorter half-life of its mRNA in the *cspC* mutant, suggesting that the main regulatory mechanism is via altering RNA stabilization. The essentiality of *cspC* for stationary phase survival may be at least in part a result of the impairment of the glyoxylate cycle.

### Availability of supporting data

The data sets supporting the results of this article are available in the Gene Expression Omnibus (GEO) repository, under accession number GSE61726 [http://www.ncbi.nlm.nih.gov/geo/query/acc.cgi?acc=GSE61726].

## Additional file

Additional file 1: Table S1. Primers used in this study. (PDF 147 kb)

## Abbreviations

cDNA: Complementary DNA synthesized from RNA
CSD: Cold Shock Domain
CSP: Cold Shock Protein
g: gravity (measurement of centrifugal force)
GEO: Gene Expression Omnibus
HPLC: High-performance liquid chromatography
OD_600_: Optical density at 600 nm
ORF: Open reading frame
rpm: Rotations per minute
ROS: Reactive oxygen species
RT-PCR: Reverse-transcription polymerase chain reaction
UTR: Untranslated region
