# Supplementary material for: CspC regulates the expression of the glyoxylate cycle genes at stationary phase in Caulobacter
Source: BMC Genomics. 2015 Aug 27;16(1):638. doi: 10.1186/s12864-015-1845-1 (PMC4551563; doi:10.1186/s12864-015-1845-1)
Supplement: Additional file 1: Table S1. — Primers used in this study. (PDF 147 kb) [file 12864_2015_1845_MOESM1_ESM.pdf]

**Additional File**

# Post-transcriptional regulation of the glyoxylate cycle *aceA* gene by CspC at stationary phase

Juliana S. Santos, Heloise Balhesteros, Carolina A.P. T. da Silva, and Marilis V. Marques

**Table S1**. Primers used in this study

| Primers | Sequence (5'–3') |
|---|---|
| CC0088 F | AGCGCGACTTTTTGGCTCAGGT |
| CC0088 R | AGGTGCGCGCAAAGTCGAAGAA |
| CC1764 F | GACAGCTCCAAGTTCACCCA |
| CC1764 R | TCCAGCCCGACAGATAGACA |
| CC3581 F | ACAGGTTTTCCCGGTTCCTG |
| CC3581 R | ATGTCGCGCCAATAGCCTTC |
| CC1402 F | TGGCCACCAATGACCTGGACTA |
| CC1402 R | CGAGATCCTTCTTGGCGTTGGT |
| CC0903 F | CGACCGGTGCGCCTCTCACT |
| CC0903 R | CGAGAGCAGACCCCCGCGAA |
| CC0682 F | AGGTCGCCGGCGCTAATATG |
| CC0682 R | AGCACCAACTCTTGCTCCCCA |
| CC1765 F | ACCTCCGCTGTGCTGAAAATCC |
| CC1765 R | GCGTAGAGGCCAAAGTCGAACA |
| CC0679 F | GCTCCGCTTTGATCTCTGCCTT |
| CC0679 R | TCGATCCGATGAACCCCAACCT |
| CC0559 F | GCAAGGCGACCTTCCAGAACAT |
| CC0559 R | ACTCGCAATACTCGTCCAGCCA |
